# Associations between homocysteine, vitamin B12, and folate and the risk of all-cause mortality in American adults with stroke

**DOI:** 10.3389/fnut.2023.1279207

**Published:** 2023-11-14

**Authors:** Panpan Zhang, Xia Xie, Yurong Zhang

**Affiliations:** Department of Neurology, The First Affiliated Hospital of Xi'an Jiaotong University, Xi'an, China

**Keywords:** stroke, all-cause mortality, homocysteine, folate, vitamin B12

## Abstract

**Objective:**

Associations between plasma homocysteine (Hcy), vitamin B12, and folate and the risk of all-cause mortality are unclear. This study aimed to examine whether plasma Hcy, vitamin B12, and folate levels independently predict the risk of all-cause mortality in American adults with stroke.

**Methods:**

Data from the United States National Health and Examination Survey (NHANES; 1999–2006) were used and linked with the latest (2019) National Death Index (NDI). Cox proportional hazards models and restricted cubic splines were used to estimate the hazard ratios (HR) and 95% confidence intervals (CI) of all-cause mortality for Hcy, folate, and B12 levels in adults with stroke. Sample weights were calculated to ensure the generalizability of the results.

**Results:**

A total of 431 participants were included (average age: 64.8 years). During a median follow-up of 10.4 years, 316 deaths occurred. Hcy was positively associated with all-cause mortality in adults with stroke (HR, 1.053; 95% CI: 1.026–1.080). Stroke patients with plasma Hcy levels in the fourth quartile had a 1.631-fold higher risk of all-cause mortality (HR, 1.631; 95% CI: 1.160–2.291) than those in the first quartile. The association between plasma Hcy and all-cause mortality was strong significant in older patients (*p* for interaction = 0.020). Plasma folate and vitamin B12 concentrations were inversely correlated with Hcy concentrations [B-value (95% CI): −0.032 (−0.056– −0.008), −0.004 (−0.007– −0.002), respectively]. No significant associations were observed between folate, vitamin B12 levels, and all-cause mortality in adults with stroke.

**Conclusion:**

Plasma Hcy levels were positively associated with all-cause mortality in older adults with stroke. Folate and vitamin B12 levels were inversely correlated with Hcy. Plasma Hcy may serve as a useful predictor in mortality risk assessment and targeted intervention in adults with stroke.

## Introduction

Stroke is the second leading cause of death worldwide (1). In the United States (US), stroke ranks fifth among all causes of death, and on average, in 2020, one person died of stroke approximately every 3 min (2). A recent study showed a 0.8% decrease in stroke mortality and a 23.8% increase in actual stroke deaths (2). Stroke imposes a great burden on families and society owing to its poor prognosis and high mortality rates. The improvement of stroke outcomes is a major global public health concern. Given the limited effective treatments for stroke, the emphasis should be on prevention, via early detection and proactive management of modifiable risk factors.

Elevated plasma homocysteine (Hcy) is one of the most easily modifiable risk factors for stroke and can be caused by deficiency of either vitamin B12 or folate (vitamin B9) (3, 4). Hcy can damage vascular structures through oxidative stress and inflammation, promote atherosclerosis, and increase the risk of stroke (5). Hcy also has a strong and direct effect on stroke severity and prognosis via neurotoxicity and increased brain damage (6). Although controversial (7, 8), clinical studies have shown that hyperhomocysteinemia (HHcy) is associated with a poor prognosis for stroke (7, 9, 10). However, whether plasma Hcy levels predict the mortality risk in stroke patients remains unclear.

Folate and vitamin B12 are the major nutritional determinants of homocysteinemia. Folate and vitamin B12 effectively reduce the Hcy concentration by participating in its metabolic pathways (4). In the United States, a folate fortification policy was implemented in 1998, which significantly increased folate levels and reduced Hcy levels in Americans (11). One study suggested that the decline in stroke-related mortality in the United States tripled after folate fortification (12). B vitamins exert a protective effect on stroke prognosis (4, 13, 14). However, some studies suggest that vitamin B12 and folate do not improve stroke prognosis (15, 16) and excessive B vitamin supplementation may increase the risk of hip fractures (17) and cancer (18). There are limited studies on the correlation between B vitamins and the long-term prognosis of adults with stroke, and the results are inconsistent with respect to B vitamin levels, comorbidities, and age (8, 19–21).

Homocysteine concentrations represent a modifiable risk factor for stroke that can be prevented and treated by B vitamin supplementation. Therefore, understanding the effects of Hcy and B vitamin levels on all-cause mortality in adults with stroke is clinically relevant. Given this context, we aimed to examine the associations between Hcy, vitamin B12, and folate and all-cause mortality in United States adults with stroke using data from the National Health and Nutrition Examination Survey (NHANES).

## Materials and methods

### Study population

This cohort study used data from the NHANES 1999–2006 and was linked to the most recent (2019) National Death Index (NDI). The NHANES is a nationally representative cross-sectional survey of the non-institutionalized United States civilians with data collected in 2-year cycles. During each cycle, the NHANES was conducted based on a stratified multistage probability sampling design and included two components: a household interview and a health examination. The health examination component consisted of medical, dental, and physiological examinations, as well as laboratory tests administered by trained medical personnel in a fully equipped mobile examination center (MEC). Additional information on the design and procedures of the NHANES are available at the Center for Disease Control and Prevention website.^1^

**Figure 1:**
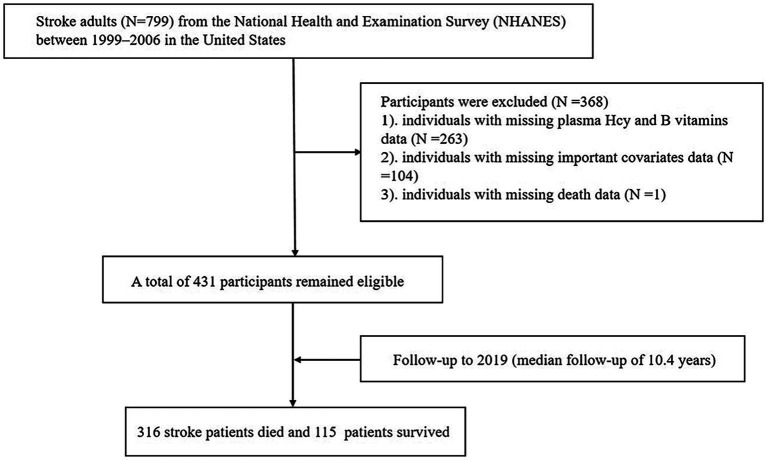
Flow chart of the study.

The study included individuals aged >20 years with stroke who participated in the 1999–2006 NHANES survey cycles. We excluded 799 participants with stroke owing to missing data on Hcy, B vitamin levels (*n* = 263) and other covariates of interest (*n* = 104), as well as those with missing mortality data (*n* = 1). The final sample consisted of 431 participants (Figure 1). All participants provided written informed consent prior to participation. The NHANES survey was approved by the Research Ethics Review Board of the National Center for Health Statistics (NCHS) and the procedures followed the principles of the Declaration of Helsinki. The NHANES data used in this study are publicly available and did not require ethical or administrative approval.

### Covariates

Sociodemographic and lifestyle information, including age, sex, race, marital status, smoking status, and educational status, were obtained from household interviews using standardized questionnaires. Race was classified as non-Hispanic white, non-Hispanic black, Mexican American, or other. Past and current smokers were defined as smokers. Non-smokers were defined as those who had never smoked. Education was defined as high school or below, and college or above. Body mass index (BMI) data and plasma Hcy, folate, and vitamin B12 levels were obtained from the MEC.

The BMI was calculated as the weight (kg)/height squared (m^2^) by trained medical personnel. Blood samples were collected in the MEC by trained laboratory technicians, and processed and transported to central laboratories following validated procedures. Plasma Hcy levels were measured using fluorescence polarization immunoassay (FPIA) method (Abbott Laboratories, Chicago, IL, United States). Serum folate and vitamin B12 concentrations were measured simultaneously by the National Center for Environmental Health at the Centers for Disease Control and Prevention using a radioprotein binding assay kit (Quantaphase II; Bio-Rad Laboratories, Hercules, CA, United States) (22, 23). History of stroke, hypertension, or diabetes was assessed using a combination of questionnaires and examination results. Stroke was defined as a diagnosis of stroke by a physician or health professional. Hypertension was defined as: (1) an average systolic blood pressure/average diastolic blood pressure ≥ 140/90 mmHg, (2) previous diagnosis by a doctor or health professional, or (3) currently being treated with antihypertensive medications. Diabetes was defined as follows: (1) based on the participants’ self-reported diagnosis of diabetes or (2) currently being treated with hypoglycemic drugs. Death outcomes were defined as the final mortality status determined by the mortality data from the NDI until December 31, 2019. Follow-up for each individual was defined as the year between the date of the NHANES interview and death, the last known survival date, or being censored in the death file. Details of these variables can be found on the official NHANES website.

### Statistical analysis

Descriptive data on participants’ baseline characteristics are expressed as weighted means and standard error (SE) or medians and interquartile ranges (IQRs) for continuous variables, and numbers and weighted percentages for categorical variables. One-way ANOVA and chi-squared tests were used to compare continuous and categorical variables, respectively.

Trends in all-cause mortality across the Hcy, folate, and vitamin 12 quartiles were tested using weighted logistic regression. Hazard ratios (HRs) and 95% confidence intervals (CIs) for all-cause mortality were assessed using weighted Cox proportional hazards models. The proportional hazard assumption by estimation of Schoenfeld’s residuals was fulfilled for Cox’s regression model. Restricted cubic splines (RCS) based on adjusted Cox proportional hazards models were used to test the linear or nonlinear associations between Hcy, B vitamins, and all-cause mortality. Weighted Kaplan–Meier plots were used to visualize the cumulative survival rate across Hcy quartiles. Linear regression analyses were used to estimate the B-values and 95% CIs for the associations between Hcy and B vitamins in adults with stroke. Two adjusted models were applied. Model 1 shows the age-adjusted results. Variables were entered in the multivariate regression models if the value of *p* was ≤0.05 in the univariate analysis. In the multivariate-adjusted Model 2, we adjusted for baseline age, sex, race, educational status, BMI, and history of diabetes.

In stratified analyses, the association between baseline Hcy and all-cause mortality was ascertained in subgroups by age (<65 and ≥ 65 years), sex (female and male), and history of hypertension (no/yes) and diabetes (no/yes) with the fully adjusted model except for stratification factors. The survey-weighted Wald test was adopted to assess the potential interaction. Data were weighted using complex survey sampling analysis methods to ensure that they were representative of United States adults. All data analyses were performed using R software (version R-4.1.0; Cary, NC, United States). Two-tailed values of *p* < 0.05 were considered statistically significant.

## Results

### Characteristics of the study population

This study included 431 adults with stroke, representative of the 3,466,111 adults with stroke in the total population. The mean age of the group was 64.8 years, and 206 participants (47.8%) were women. Among the 431 participants, the weighted mortality rate was 66.3% (316) in 2019.

Table 1 presents the characteristics of the participants stratified according to their survival state. Compared with those who were still alive, participants who had died were more likely to be older, male, non-Hispanic white, have a lower BMI, have a history of diabetes, and have higher plasma folate and Hcy concentrations. Additionally, they were less likely to be educated. There were no significant differences in the distribution of vitamin B12 concentrations, marital status, smoking status, or history of hypertension.

**Table 1:** Baseline characteristics of all included participants by survival state from NHANES 1999–2006.

Characteristic	Total (*n* = 431)	Alive (*n* = 115)	Death (*n* = 316)	*p* value
Age, in years^a^	64.77 (1.07)	51.89 (1.47)	71.32 (0.85)	< 0.001
Female, *n* (%)	206 (47.80)	76 (70.95)	130 (51.78)	0.010
Race, *n* (%)				0.009
Non-Hispanic White	252 (58.47)	47 (65.45)	205 (82.92)	
Non-Hispanic Black	84 (19.49)	32 (15.20)	52 (9.18)	
Mexican American	72 (16.71)	26 (6.15)	46 (3.12)	
Other Race	23 (5.34)	10 (13.20)	13 (4.78)	
Education status, *n* (%)				< 0.001
College or above	150 (34.8)	49 (58.52)	101 (34.12)	
High school or below	281 (65.2)	66 (41.48)	215 (65.88)	
Smoking status, *n* (%)				0.093
Former	168 (38.98)	35 (24.17)	133 (38.07)	
Never	186 (43.16)	53 (45.47)	133 (42.68)	
Now	77 (17.87)	27 (30.36)	50 (19.25)	
Marital status, *n* (%)				0.205
Married/Living with partner	239 (55.45)	65 (57.16)	174 (55.27)	
Never married	21 (4.87)	10 (9.69)	11 (4.38)	
Widowed/Divorced/Separated	171 (39.68)	40 (33.15)	131 (40.4)	
Body mass index (kg/m^2^)^a^	29.78 (0.39)	31.00 (0.70)	29.16 (0.46)	0.031
Homocysteine (μmol/L)^b^	11.44 (0.32)	9.75 (0.68)	12.31 (0.41)	0.004
Folate (nmol/L)^b^	39.98 (2.11)	33.39 (2.86)	43.34 (2.41)	0.005
Vitamin B12 (pmol/L)^b^	408.51 (15.58)	414.92 (27.10)	405.24 (19.07)	0.771
Medical history, *n* (%)				
Diabetes	133 (30.86)	25 (17.11)	108 (33.30)	0.010
Hypertension	351 (81.44)	87 (72.73)	264 (81.56)	0.134

^a^Mean (SE); ^b^Median (interquartile ranges).

### Associations between Hcy, B vitamins, and all-cause mortality

Table 2 shows that the plasma Hcy concentration (both as categorized and as continuous variables) were positively correlated with all-cause mortality in adults with stroke. A significant correlation was observed between plasma Hcy as a continuous variable and risk of all-cause mortality (multivariate-adjusted HR 1.053, 95% CI 1.026–1.080, *p* < 0.001). A one-unit increase in plasma Hcy level was associated with a 5.3% higher risk of death in adults with stroke. There was no significant continuous correlation between serum folate, vitamin B12, and all-cause mortality.

**Table 2:** Associations of homocysteine and B vitamins with all-cause mortality in adults with stroke.

Variables	Mortality	Model 1 HR (95%CI)	*p* value	Model 2 HR (95% CI)	*p* value	*p* for non-linearity
*Continuous*						
*Homocysteine*	316/431	1.057 (1.034, 1.081)	<0.001	1.053 (1.026, 1.080)	<0.001	>0.05
*Folate*	316/431	1.003 (0.999, 1.007)	0.156	1.002 (0.998, 1.006)	0.374	>0.05
*Vitamin B12*	316/431	1.000 (1.000, 1.001)	0.329	1.000 (1.000, 1.001)	0.092	>0.05
*Categorical*						
*Homocysteine*						
Quartile1 (<8.5)	50/108	reference		reference		
Quartile2 (8.5,10.8)	83/109	1.314 (0.940, 1.838)	0.110	1.123 (0.781, 1.615)	0.532	
Quartile3 (10.8,13.3)	88/106	1.969 (1.385, 2.798)	<0.001	1.750 (1.197, 2.558)	0.004	
Quartile4 (>13.3)	95/108	1.919 (1.407, 2.617)	<0.001	1.631 (1.160, 2.291)	0.005	
*p* for trend		<0.001		<0.001		
*Folate*						
Quartile1 (<21.2)	70/108	reference		reference		
Quartile2 (21.2,30.8)	74//108	0.931 (0.673, 1.287)	0.665	1.000 (0.710, 1.408)	0.998	
Quartile3 (30.8,51.1)	81/107	0.660 (0.446, 0.976)	0.038	0.726 (0.494, 1.067)	0.103	
Quartile4 (>51.1)	91/108	0.994 (0.647, 1.527)	0.978	0.971 (0.670, 1.408)	0.878	
*p* for trend		0.156		0.374		
*Vitamin B12*						
Quartile1 (<248.0)	83/109	reference		reference		
Quartile2 (248.0,332.8)	77/108	0.873 (0.584, 1.307)	0.510	1.010 (0.669, 1.525)	0.963	
Quartile3 (332.8,475.3)	79/106	0.919 (0.618, 1.368)	0.678	1.079 (0.678, 1.717)	0.749	
Quartile4 (>475.3)	77/108	0.909 (0.595, 1.389)	0.660	1.086 (0.730, 1.616)	0.682	
*p* for trend		0.329		0.092		

Model 1: Adjusted for age.

Model 2: Adjusted for age, sex, race, educational status, body mass index, and history of diabetes.

Using the Hcy level as the categorical variable, participants in the highest quartile of Hcy had a 63.1% higher risk of all-cause mortality than those in the lowest quartile (multivariate-adjusted HR 1.631, 95% CI 1.160–2.291, *p* for trend <0.001). We also found no significant correlation between the quartiles of folate, vitamin B12, and risk of all-cause mortality in adults with stroke. The adjusted HRs (95% CIs) from the bottom to the top quartile of serum folate and folate for all-cause mortality were 1.00 (reference), 1.00 (0.710–1.408), 0.726 (0.494–1.067), and 0.971 (0.670–1.408; *p* for trend = 0.374) and 1.00 (reference), 1.010 (0.669–1.525), 1.079 (0.678–1.717), and 1.086 (0.730–1.616; *p* for trend = 0.092), respectively. Restricted cubic splines showed the linear relationship between Hcy, folate, vitamin B12, and all-cause mortality risk in adults with stroke (*p* for non-linearity > 0.05).

**Figure 2:**
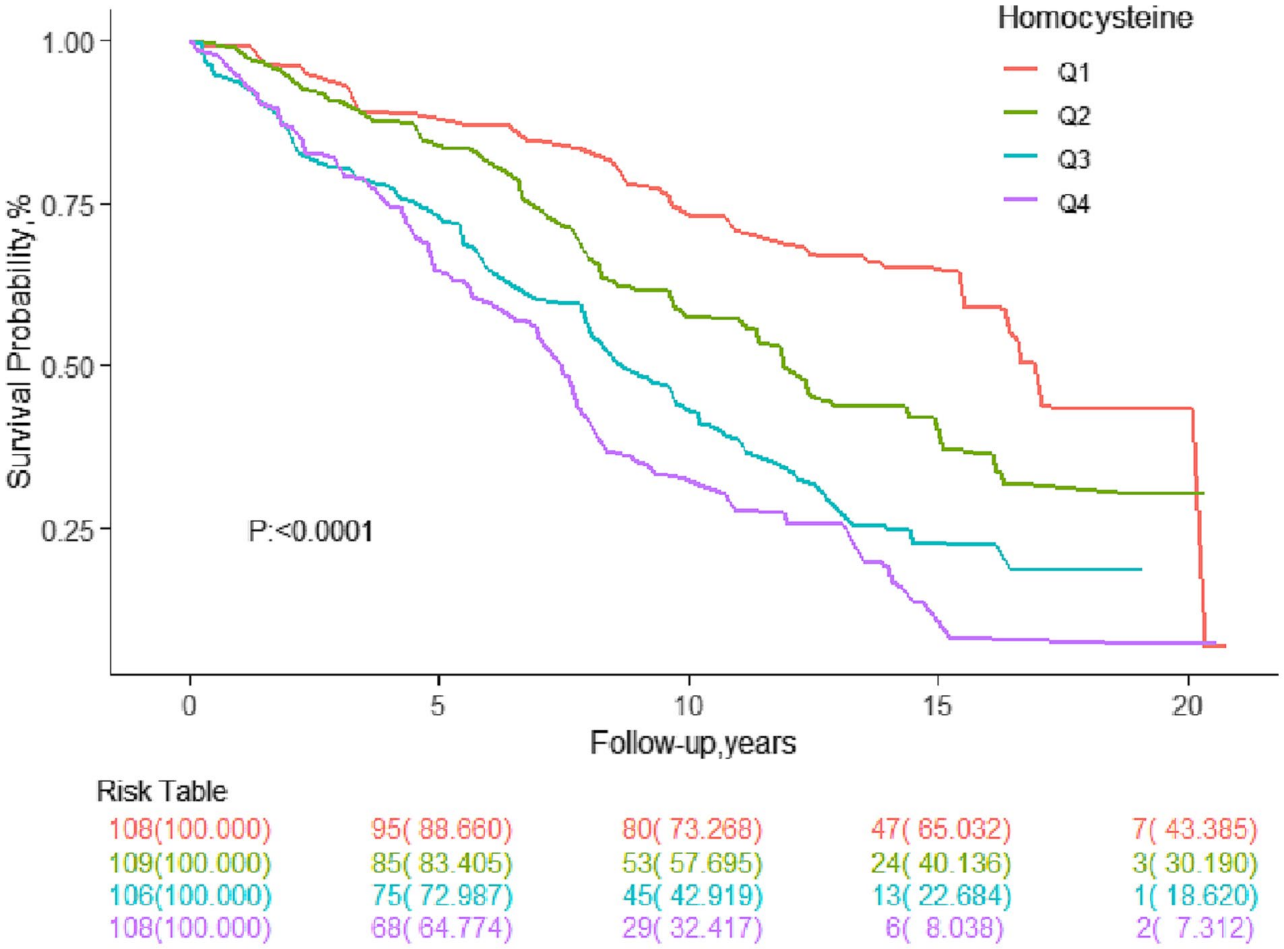
Survival curve of the stroke population.

Figure 2 presents Kaplan–Meier curves for the cumulative survival rate stratified by Hcy quartiles. The higher the Hcy level of adults with stroke, the lower their survival rate.

### Correlations between folate, vitamin B12, and Hcy levels

Table 3 shows the results of the multivariate linear regression analysis of serum folate, vitamin B12, and Hcy levels. Serum folate and vitamin B12 levels are inversely correlated with plasma Hcy levels. The multi-adjusted B-values (95% CI) of Hcy for folate and vitamin B12 were −0.032 (−0.056 to −0.008; *p* = 0.011) and −0.004(−0.007 to −0.002; *p* = 0.002), respectively.

**Table 3:** Associations of homocysteine with B vitamins in adults with stroke.

Variables	Model 1	*p* value	Model 2	*p* value
	*B*-value (95% CI)		*B*-value (95% CI)	
*Folate*	−0.031 (−0.054, −0.008)	0.010	−0.032 (−0.056, −0.008)	0.011
*Vitamin B12*	−0.004 (−0.007, −0.002)	0.002	−0.004 (−0.007, −0.002)	0.002

Model 1: Adjusted for age.

Model 2: Adjusted for age, sex, race, education status, body mass index, and history of diabetes.

### Stratification analysis

**Figure 3:**
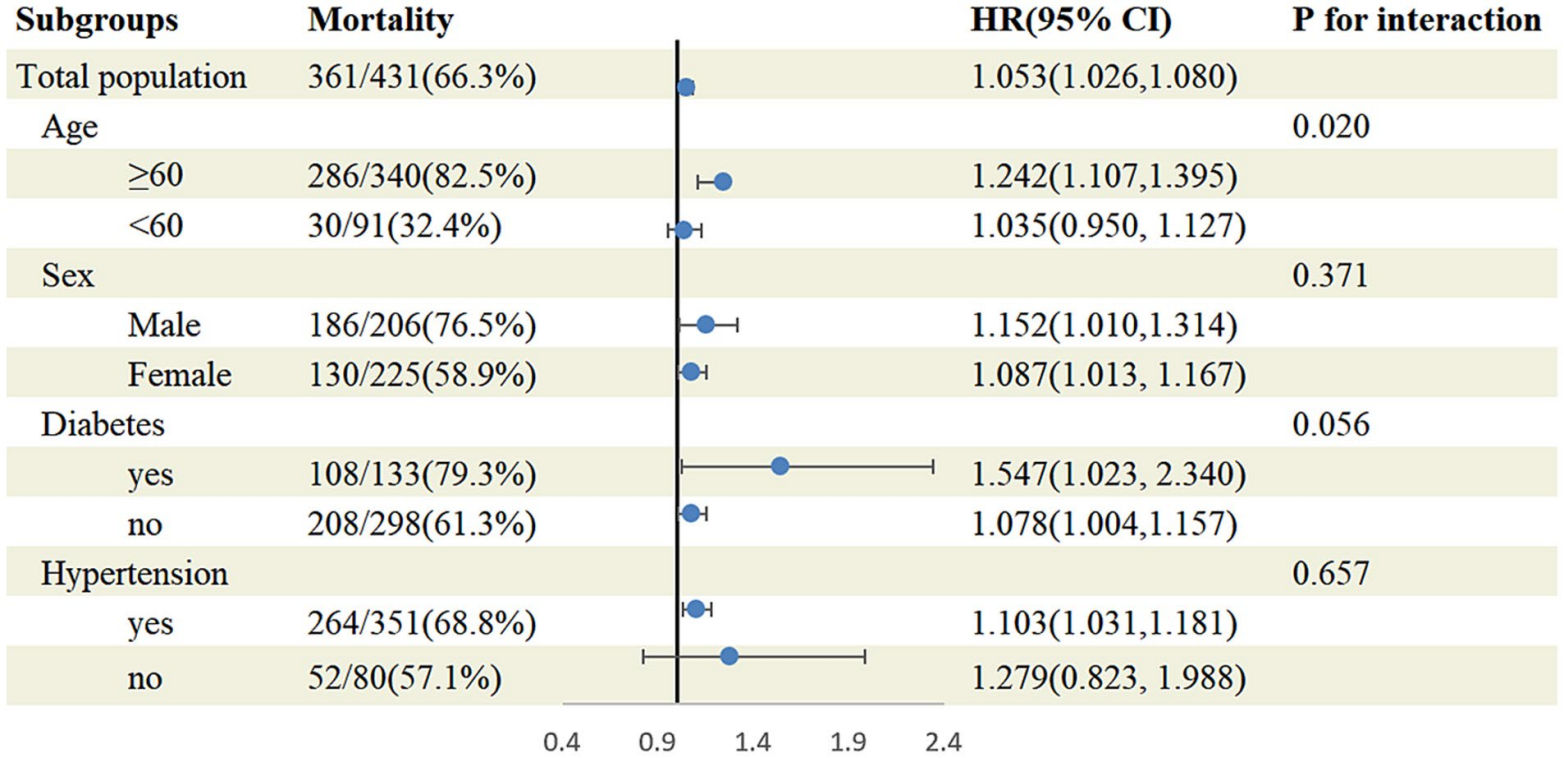
Subgroup analysis of the association between homocysteine and all-cause mortality. Results were adjusted for all covariates except the corresponding stratification variable.

In stratified analyses (Figure 3), the association of plasma Hcy with increased risk of all-cause mortality was largely consistent in most subgroups. As shown in the forest plot, no interactions were observed between plasma Hcy concentration and sex (*p* for interaction = 0.371), diabetes (*p* for interaction = 0.056), or hypertension (*p* for interaction = 0.657); therefore, none of these variables significantly modified the association between Hcy levels and all-cause mortality in adults with stroke. However, Hcy significantly increased the risk of all-cause mortality in older adults with stroke, and age and Hcy had an interaction effect (*p* for interaction = 0.020).

## Discussion

In a representative United States cohort of adults with stroke with a median follow-up of 10.4 years, we found that higher plasma Hcy levels were significantly associated with lower long-term survival in older adults with stroke; however, no significant association was observed between folate, vitamin B12, and all-cause mortality. In addition, higher folate and vitamin B12 levels were associated with lower Hcy levels. These findings suggest that plasma Hcy levels might be a useful indicator for assessing risk of all-cause mortality in clinical practice.

The association between Hcy and all-cause mortality risk in adults with stroke has not been well established. Our study found a linear and positive correlation between Hcy and all-cause mortality in adults with stroke. Every 1 μmol/L increase in Hcy concentration increased mortality in these patients by 5.3%. Data specific to the stroke population are unavailable; however, previous studies of the general, non-stroke population have reported a higher all-cause mortality risk with high plasma Hcy levels (24, 25). Many previous studies have reported the effect of Hcy levels on stroke prognosis and the results have been controversial (7, 9, 20, 26, 27). A 16.4- and 6-year follow-up study in Norway found that Hcy significantly increased long-term stroke mortality (20, 26). However, in a 2-year cohort study, a moderate reduction in total Hcy in patients with non-disabling cerebral infarction had no effect on vascular outcomes (8). A Chinese stroke study showed that Hcy was significantly associated with poor functional outcomes at discharge but not with in-hospital mortality in patients with spontaneous intracerebral hemorrhage (10). However, most studies have focused on the short-term prognosis of patients with stroke. Evidence shows that the risk factors for long- and short-term mortality from stroke differ partially (26). In addition, it should be noted that after the introduction of the folate fortification program in the United States, the Hcy level in the United States population was significantly reduced (11) and the mortality rate for stroke was reduced by a factor of 3 (12). Stroke is the second leading cause of death worldwide (1), but ranks fifth among all causes of death in the United States population (2), which indicates that the distribution of Hcy concentration and death composition differ in different regions. Therefore, different population backgrounds are likely to influence the effect of Hcy on mortality in adults with stroke. Further studies are needed to indicate the underlying biological mechanisms.

We further stratified our analysis by age, sex, hypertension, and diabetes to explore the relationship between Hcy and all-cause mortality in different settings among United States adults with stroke. Higher levels of Hcy were found to increase the risk of mortality only in the association between age and Hcy (*p*-interaction = 0.020), indicating that Hcy is a strong risk factor for all-cause mortality in older adults with stroke. In previous studies, old age, male, history of hypertension, and diabetes were found to be risk factors for stroke (2, 28) and had a synergistic effect on stroke with Hcy (29, 30). Screening for risk factors in high-risk populations to develop more personalized Hcy treatment strategies, as well as controlling traditional risk factors, such as blood pressure, blood glucose, and smoking status, are important for improving stroke outcomes. Our analysis adds to the literature by showing that age could greatly increase the adverse effects of Hcy on all-cause mortality in adults with stroke. It is unclear why higher Hcy concentrations would be associated with higher all-cause mortality risk among adults with stroke, especially in older adults. A possible explanation is that B vitamins are the major nutritional determinants of Hcy levels, and their dietary deficit, along with a physiological age-related reduction in renal function, is responsible for most cases of HHcy in older adults (31, 32).

No association was observed between serum folate and long-term mortality in adults with stroke in our study. In this study, participants underwent folate fortification, which generally improves blood folate levels (11). One study has shown that low-dose (0.4 mg/day) folate supplementation is sufficient to improve vascular endothelial function, while increasing the dose to 5 mg/day has no additional benefit (33). This may be why our study suggests that further folate increases did not reduce mortality in adults with stroke who had already received folate fortification. The effects of folate on mortality are inconsistent in different populations. In the general population included in the NHANES from 1999 to 2010, Peng et al. (34) found that low folate levels were significantly associated with a higher risk of all-cause mortality. In the population with cardiovascular diseases, folate treatment was not significantly associated with all-cause mortality (21). In a hemodialysis population, folate supplementation reduced total mortality (19). However, Leung et al. (35) found that neither low-nor high-dose folate intake was significantly associated with stroke mortality in individuals with kidney disease. These studies suggest that the association between folate levels and mortality may be influenced by disease background and causes of death.

No protective effects of vitamin B12 on long-term mortality in adults with stroke were observed in this study. The association between vitamin B12 levels and mortality rates is inconsistent. A NHANES study showed that low serum vitamin B12 levels were associated with increased all-cause mortality in the general population (36). Mendonça et al. (37) found that higher vitamin B12 levels were associated with a higher risk of all-cause and cardiovascular mortality in women. Consistent to our results, there was no association of vitamin B12 levels with all-cause mortality was found in older people in the United Kingdom (38). The United States Preventive Services Task Force concluded that the current evidence to assess the balance of benefits and harms of using multivitamin supplements to prevent cardiovascular disease is insufficient (39). Measurements of vitamin B12 was based on a single B12 concentration at baseline and its high intra-individual variability, are likely to underestimate the association with mortality due to regression dilution bias in our cohort as well as in others (38).

In this study, we explored the correlations between folate, vitamin B12, and Hcy levels in adults with stroke. Consistent with a previous study (4), folate and vitamin B12 levels were inversely correlated with Hcy levels, indicating that supplementation with folate and vitamin B12 can reduce Hcy levels. A few studies have shown that folate and vitamin B12 indirectly reduce the risk of stroke (40) and death (41, 42) by reducing the Hcy levels. Further research is required to explore the impact of interventions targeting Hcy levels using folate and vitamin B6 supplementation on mortality in stroke patients.

Our results extend the previously reported associations of Hcy with all-cause mortality and the absence of associations between folate and vitamin B12 with all-cause mortality in the older stroke population. These results highlight the potential advantages of monitoring and evaluating Hcy status in the prevention of all-cause mortality for adults with stroke. In addition, the strengths of this study are its prospective design, long-term follow-up period, and ascertainment of mortality by validated NDI data.

Despite its critical findings, this study has a few limitations. First, causality cannot be concluded due to the observational study design. The genetic variants in Hcy metabolism-related genes may provide more information for the causal relationship between Hcy and outcomes. Second, stroke was self-reported, which is likely to introduce bias. However, this questionnaire has been widely used in studies assessing self-reported stroke. Third, although this study accounted for major covariates, the possibility of residual confounding factors cannot be excluded. Fourth, measurements of plasma Hcy, folate, and vitamin B12 were based on single blood samples. This is likely to underestimate the associations with mortality due to regression dilution bias. Finally, the relationships explored in this study were based on United States adults (1999–2006 NHANES data), a country where folate fortification is administered; therefore, caution must be taken when generalizing these findings to populations without folate fortification. Further studies are required to explore the relationship between plasma Hcy and B vitamins and long-term mortality in adults with stroke among different populations. Additional analyses are needed to examine Hcy-lowering over the course of the trial on all-cause mortality in adults with stroke.

## Conclusion

In this prospective American study, plasma Hcy was linearly and positively associated with the risk of all-cause mortality in older adults with stroke. Folate and vitamin B12 levels were inversely correlated with Hcy levels but had no effect on long-term survival in adults with stroke. Further research are needed to indicate the potential mechanisms underlying the observed associations and the impact of interventions targeting Hcy levels by folate and vitamin B6 supplementation in stroke patients.

## Data availability statement

The datasets presented in this study can be found in online repositories. The names of the repository/repositories and accession number(s) can be found at: https://wwwn.cdc.gov/nchs/nhanes/.

## Ethics statement

The studies involving humans were approved by the Research Ethics Review Board of the National Center for Health Statistics (NCHS). The studies were conducted in accordance with the local legislation and institutional requirements. The participants provided their written informed consent to participate in this study.

## Author contributions

PZ: Conceptualization, Data curation, Formal analysis, Investigation, Methodology, Resources, Software, Validation, Writing – original draft. XX: Investigation, Validation, Writing – review & editing. YZ: Conceptualization, Formal analysis, Funding acquisition, Methodology, Supervision, Visualization, Writing – review & editing.

## Footnotes

^1^https://wwwn.cdc.gov/nchs/nhanes
